# Analysis of VSV pseudotype virus infection mediated by rubella virus envelope proteins

**DOI:** 10.1038/s41598-017-10865-2

**Published:** 2017-09-14

**Authors:** Masafumi Sakata, Hideki Tani, Masaki Anraku, Michiyo Kataoka, Noriyo Nagata, Fumio Seki, Maino Tahara, Noriyuki Otsuki, Kiyoko Okamoto, Makoto Takeda, Yoshio Mori

**Affiliations:** 1 Department of Virology 3, National Institute of Infectious Diseases, 4-7-1 Gakuen, Musashimurayama-shi, Tokyo 208-0011 Japan; 2 Department of Virology 1, National Institute of Infectious Diseases, 4-7-1 Gakuen, Musashimurayama-shi, Tokyo 208-0011 Japan; 3 Department of Microbiology, Yokohama City University School of Medicine, 3-9 Fukuura Yokohama Kanazawa-ku, Kanagawa, 236-0004 Japan; 4 Department of Pathology, National Institute of Infectious Diseases, 4-7-1 Gakuen, Musashimurayama-shi, Tokyo 208-0011 Japan; 5 Present Address: Department of Virology, University of Toyama, 2630 Sugitani Toyama-shi, Toyama, 930-0194 Japan

## Abstract

Rubella virus (RV) generally causes a systemic infection in humans. Viral cell tropism is a key determinant of viral pathogenesis, but the tropism of RV is currently poorly understood. We analyzed various human cell lines and determined that RV only establishes an infection efficiently in particular non-immune cell lines. To establish an infection the host cells must be susceptible and permissible. To assess the susceptibility of individual cell lines, we generated a pseudotype vesicular stomatitis virus bearing RV envelope proteins (VSV-RV/CE2E1). VSV-RV/CE2E1 entered cells in an RV envelope protein-dependent manner, and thus the infection was neutralized completely by an RV-specific antibody. The infection was Ca^2+^-dependent and inhibited by endosomal acidification inhibitors, further confirming the dependency on RV envelope proteins for the VSV-RV/CE2E1 infection. Human non-immune cell lines were mostly susceptible to VSV-RV/CE2E1, while immune cell lines were much less susceptible than non-immune cell lines. However, susceptibility of immune cells to VSV-RV/CE2E1 was increased upon stimulation of these cells. Our data therefore suggest that immune cells are generally less susceptible to RV infection than non-immune cells, but the susceptibility of immune cells is enhanced upon stimulation.

## Introduction

Rubella is an acute infectious viral disease characterized by low-grade fever, a short-lived morbilliform rash, and lymphadenopathy^1^. Additionally, arthritis often develops in rubella patients, particularly in adolescents and adult female patients, and encephalitis, while rare, is a severe complication of this disease. Most importantly, neonates born from mothers who suffered from rubella during the first trimester of pregnancy may develop congenital rubella syndrome (CRS) and multiple organ malformations. Congenital cataracts, sensorineural hearing loss, and cardiovascular defects are most common in CRS.

Rubella virus (RV), the etiologic agent of rubella and CRS, belongs to the genus *Rubivirus* in the *Togaviridae* family. Despite the great importance of RV to public health, the molecular mechanisms underlying RV pathogenicity remain poorly understood. Only humans are the natural hosts for RV, but cell lines from monkeys, hamsters and rabbits such as Vero, BHK, and RK-13, respectively, are commonly used for isolation or propagation of RV, because RV replicates most efficiently in these cell lines^2–4^. Understanding the cell types targeted by RV and the molecular basis for determining viral tropism is an important step for understanding the pathophysiology of rubella and CRS. Myelin oligodendrocyte glycoprotein (MOG) has been recently identified as a receptor for RV^5^. However, MOG is expressed mainly in cells of central nervous system, and its expression is very low or undetectable in the cells from other organs or tissues. Since RV generally causes a systemic infection, the pathology of rubella and CRS cannot be simply explained by the distribution pattern of MOG. Previous studies have indicated that membrane phospholipids and glycolipids, rather than cellular surface proteins, support RV infection, suggesting a functional role for membrane lipids in RV infections^6, 7^.

Vesicular stomatitis virus (VSV) belongs to the *genus Vesiculovirus* in the *Rhabdoviridae* family, and the genome is a non-segmented negative-sense RNA. A reverse genetics system for VSV has been previously established, permitting to engineer the infectious VSV genome^8, 9^. Recombinant VSVs, in which authentic glycoprotein, G protein, gene is replaced with a reporter protein gene such as a fluorescent protein, luciferase, or secreted alkaline phosphatase, can normally bud from cells even in the absence of G protein^10–14^. Envelope proteins of different virus species can be incorporated into VSV particles, even when they are provided *in trans*, generating pseudotype VSVs. Pseudotype VSVs enter cells through receptor binding and membrane fusion mediated by incorporated envelope proteins of different viruses, and the entry can be traced with reporter gene expression in the recombinant VSV genome^15–17^.

In the present study, various human cell lines were tested for their susceptibility (defined as the capacity for viral entry) to RV using a recombinant VSV pseudotyped with RV proteins.

## Results

### RV only establishes an efficient infection in specific non-immune cell lines

**Figure 1:**
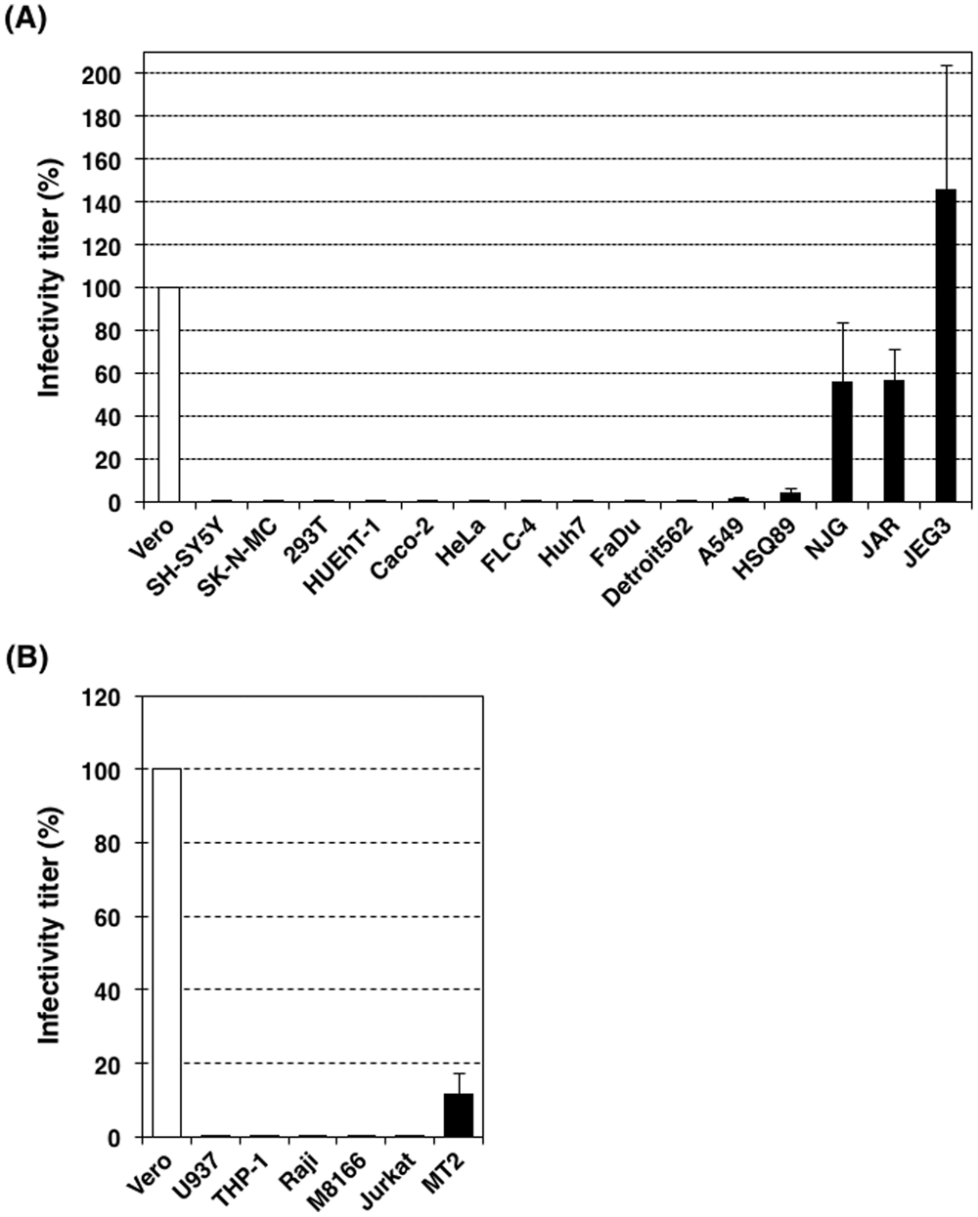
Infectivity titers of RV in various cell lines. The infectivity titer (50% cell culture infectious dose) of a stock solution of RV was determined using Vero and various human cell lines. The infectivity titer determined using Vero cells was set to 100%. (**A**) Data using non-immune cell lines. (**B**) Data using immune cell lines. Data represent the mean values ± standard deviation (SD) of three independent experiments.

Measuring the infectivity titer of a virus is fundamental in virus research. However, infectivity titers are not absolute values and are dependent on the host cells used to determine such titers. The host cells must be both susceptible and permissible for virus infection establishment. Therefore, even when the viral titer of a particular sample is measured in different cell lines, it may differ greatly. In this experiment, we have determined the 50% cell culture infectious dose (CCID_50_) by using a limiting dilution method, in which multiple rounds of infection is allowed. However, the CCID_50_ in this method is basically expected to show the first cell numbers infected with RV. Indeed, even when the second round of infection was blocked by NH_4_Cl, similar results were obtained (Supplementary Figure 1). The infectivity titer of a stock of the recombinant RV rHS strain was determined using various human cell lines and a monkey kidney-derived cell line, Vero. Vero cells are most commonly used to determine the infectivity titer of RV. The infectivity titer of the rHS stock was 5.17 × 10^6^ CCID_50_/cell when Vero cells were used. The titer was set to 100% to simplify comparison (Fig. 1A, B). When NJG, JAR and JEG3 cells were used, the infectivity titers of the same stock of rHS were 56.1%, 56.7%, and 146%, respectively (Fig. 1A). Therefore, the three trophoblast-derived cell lines were susceptible and permissible to RV infection similarly to Vero cells. In contrast, the titers were reduced to only ~4 and 18%, respectively, when HSQ89 and A549 cells were used (Fig. 1A). The titers were very low (~0.01 to 0.1%), when the other ten non-immune cell lines (SH–SY5Y, SK–N–MC, 293T, HUEhT–1, Caco–2, HeLa, FLC–4, Huh7, FaDu, and Detroit562) were used (Fig. 1A). Therefore, these cell lines are almost non-susceptible or non-permissible, or both, to RV infection. The titers of the viral stock were also determined using immune cell lines. The titer was ~12% when MT2 cells were used, whereas the titers were very low (~0.01 to 0.1%) when the other five immune cell lines (U937, THP-1, Raji, M8166, and Jurkat) were tested (Fig. 1B). Therefore, RV infection is established efficiently in only some non-immune cell lines, particularly trophoblast-derived cell lines.

### Generation of a pseudotype VSV bearing RV envelope proteins

**Figure 2:**
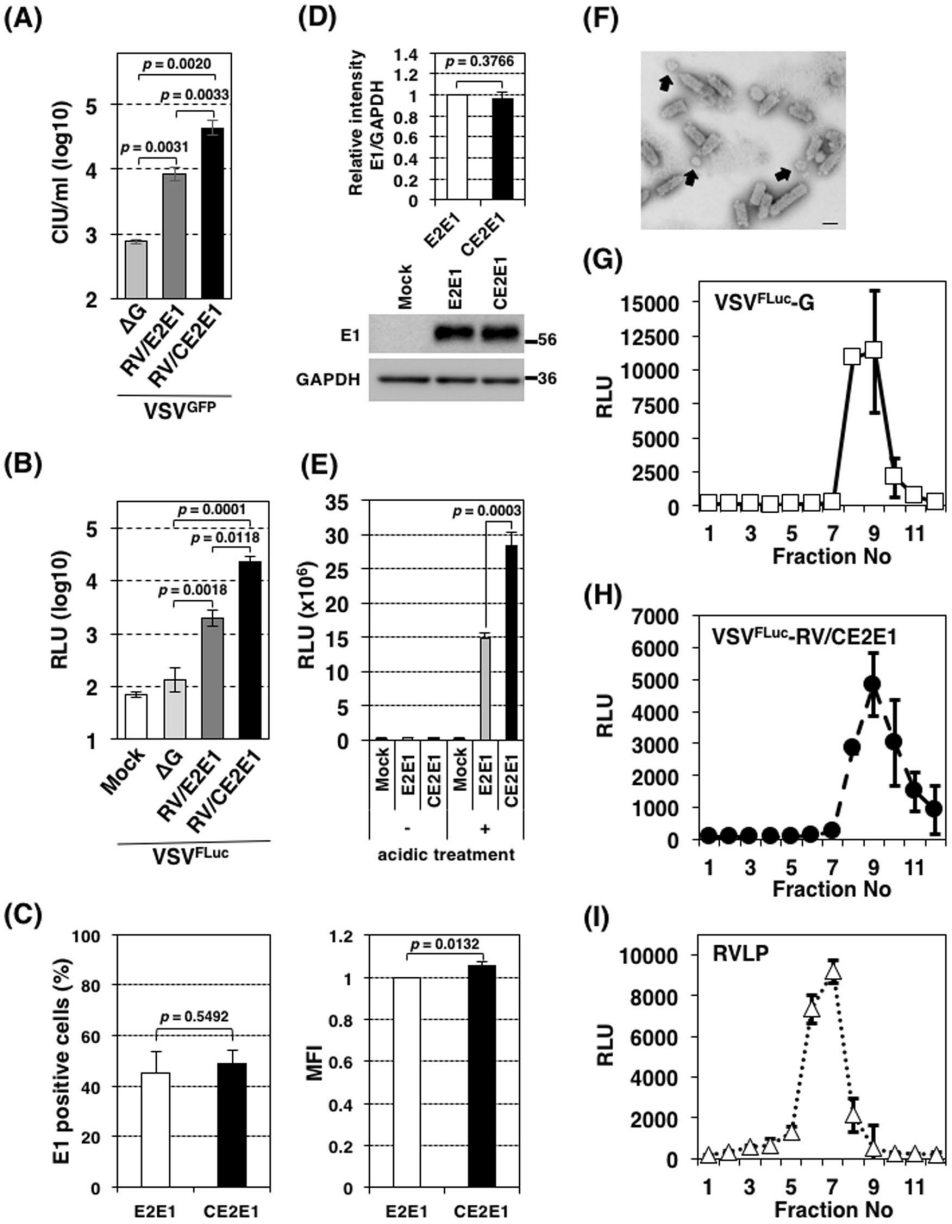
Properties of the VSV-RV/CE2E1 pseudotype virus. (**A**) Infectivity of VSV^GFP^-RV/E2E1, VSV^GFP^-RV/CE2E1, and VSV^GFP^-∆G. Vero cells were infected with VSV^GFP^-RV/E2E1, VSV^GFP^-RV/CE2E1 or VSV^GFP^-∆G, and at 24 h post-infection (p.i.) the cell infectious units (CIUs) were determined by counting the number of GFP-expressing cells. (**B**) Infectivity of VSV^FLuc^-RV/E2E1, VSV^FLuc^-RV/CE2E1, and VSV^FLuc^-∆G. Vero cells were infected with VSV^FLuc^-RV/E2E1, VSV^FLuc^-RV/CE2E1, or VSV^FLuc^-∆G, and at 24 h p.i. the luciferase activities in the cells were measured.(**C**) Effect of the C protein on the cell surface expression of the E1 protein. Expression of the E1 protein on the surface of 293 T cells transfected with pcDNA3.1-E2E1 or pcDNA3.1-CE2E1 were detected by flow cytometry using anti-RV E1 antibody. The left panel shows percentage of cells expressing the E1 protein on the cell surface, and the right panel shows expression level of the E1 protein in the positive cells indicated as a mean fluorescent intensity (MFI). (**D**, **E**) The total amount of the E1 protein in cells co-expressed with or without the C protein. 293CD4/DSP_1–7_ and 293FT/DSP_8–11_ cells constitutively expressing DSP_1–7_ and DSP_8–11_, respectively, were mixed and cultured together. The cells were transfected with pcDNA3.1-E2E1, pcDNA3.1-CE2E1 or the empty vector and incubated for 32 hours. (**D**) The cell lysates were subjected to immunoblotting with anti-RV E1 and anti–GAPDH antibodies. The signal intensity of the E1 protein in the cells was normalized by those of GAPDH. The full-length images are shown in Supplementary Fig. 2. (**E**) The cells were incubated with low pH (pH 5.1) media for 15 min and then were cultured with the standard culture media for 8 h. The *Renilla* luciferase activity derived from fusion cells were measured and normalized by expression levels of the E1 protein determined in (**D**). (**F**) Electron microscope image of particles in the VSV-RV/CE2E1 stock solution. Purified virions in the VSV^GFP^-RV/CE2E1 stock solution were fixed with 2% paraformaldehyde, and then were negatively stained with 2% phosphotungstic acid solution. The arrows indicate spherical particles. Bar indicates 100 nm. (**G**, **H**, **I**) Viral particle densities. VSV^FLuc^-G (**G**), VSV^FLuc^-RV/CE2E1 (**H**), and RV virus like-particles (RVLPs) (**I**) were fractionated in a sucrose-gradient by ultracentrifugation. Twelve fractions were collected from top to bottom of the gradient. VSV^FLuc^-G, VSV^FLuc^-RV/CE2E1, and RVLP were detected by infecting Vero cells with individual fractions from the gradient and measuring the luciferase activities in the cells. (**B**, **E G**–**I**) RLU, relative light unit of luciferase activity. (**A**-**E**, **G**-**I**) Data represent the mean values ± standard deviations for triplicate samples. (**A**-**E**) The significant differences were determined by two-tailed *t*-tests.

Data from the experiments with RV indicated that many cell lines were nearly non-susceptible or non-permissible, or both, to RV infection. To assess the susceptibilities of individual cell lines to infection with RV, pseudotype VSVs bearing RV envelope proteins were generated. The VSV pseudotype virus genomes lack the G gene, which is replaced with a reporter protein gene, the green fluorescent protein (GFP) or firefly luciferase (FLuc) gene^8, 14, 18, 19^. The pseudotype viruses were thus expected to show similar behaviors to RV during the process of virus entry, and the subsequent processes of gene expression are dependent on the VSV replication machinery. In the initial experiment, only RV envelope E2 and E1 proteins were provided in *trans* to generate the GFP gene- and FLuc gene-encoding pseudotype viruses, VSV^GFP^-RV/E2E1 and VSV^FLuc^-RV/E2E1, respectively, like other VSV pseudotype viruses^13, 19–30^. The infectivity titers for VSV^GFP^-RV/E2E1 and VSV^FLuc^-RV/E2E1 were 10-fold higher than those of the counterpart control viruses, VSV^GFP^-∆G and VSV^FLuc^-∆G, respectively, which lack envelope glycoproteins, in Vero cells (Fig. 2A, B). Although this suggests that RV envelope proteins contribute to the infectivity of the pseudotype viruses, they seem to have little practical application because of their low infective titers. Co-expression of the Capsid (C) protein resulted in production of the pseudotype viruses, VSV^GFP^-RV/CE2E1 and VSV^FLuc^-RV/CE2E1 and these pseudotype viruses showed higher infectivity titers than VSV^GFP^-RV/E2E1 and VSV^FLuc^-RV/E2E1, respectively (Fig. 2A, B). The infectivity titers for VSV^GFP^-RV/CE2E1 and VSV^FLuc^-RV/CE2E1 were 50–200-fold higher than those of VSV^GFP^-∆G and VSV^FLuc^-∆G, respectively. An experiment indicated that the RV C protein promotes fusion activity in RV envelope (E1 and E2) proteins by supporting the maturation or stabilizing either E2 and E1 or their interactions during intracellular transport to the cell surface^31^. We have confirmed the enhance effect by the C protein on fusion by RV envelope proteins. The surface expression level of the E1 protein with the C protein was similar to that without the C protein (Fig. 2C). The total amounts of the E1 protein in cells were also similar between cells co-expressed with or without the C protein (Fig. 2D). Nevertheless, the level of cell-to-cell fusion was increased by ~two-fold by co-expressing the C protein (Fig. 2E). Although the detailed mechanism was unclear, the data demonstrated that the RV envelope protein expressed on the cell surface showed a better fusion activity than that expressed without the C protein. Thus, in the following experiment, the C protein was provided together with RV envelope E2 and E1 proteins. However, it should be noted that co-expression of the C protein with the E1 and E2 proteins may produce empty non-infectious RV-like-particles (RVLP). Indeed, an electron microscopic assay revealed both bullet-shaped (~61 ± 7 nm × 173 ± 18 nm; n = 5) and spherical particles (~73 ± 12 nm in diameter; n = 8), which were corresponding to VSV and RV virions, respectively^9, 32^. This suggests that pseudotype VSV and RVLP were contained in the VSV^GFP^-RV/CE2E1 stocks (Fig. 2F). No particles showed a combined shape of the bullet and spherical forms. Although the VSV pseudotype virus genome was expected to be incorporated into the bullet shaped particles, it was confirmed by the following experiment. Stock solutions of VSV^FLuc^-G, VSV^FLuc^-RV/CE2E1 and RVLP were independently prepared and concentrated and fractionated by sucrose-gradient ultracentrifugation. In this experiment, RVLP, which contains a subgenomic replicon RNA in which the structural genes are replaced with DNA sequences encoding the puromycin *N*-acetyl-transferase protein, the foot-and-mouth disease virus 2 A self-cleavage domain, and the *Renilla* luciferase^33^, was used as a marker for RVLP fractionation pattern. VSV^FLuc^-G, VSV^FLuc^-RV/CE2E1, and RVLP in individual fractions were detected by infecting Vero cells with the fractions and measuring the relative light unit (RLU) from the cells. The peak infectivity of VSV^FLuc^-RV/CE2E1 was at ninth fraction similarly to that of VSV^FLuc^-G, with no peak or shoulder at seventh fraction where RVLP indicated the peak infectivity (Fig. 2G-I). The profiles demonstrated that the pseudotype VSV particles, but not RVLP, incorporated the genome.

### VSV-RV/CE2E1 undergoes a similar entry process to the authentic RV

**Figure 3:**
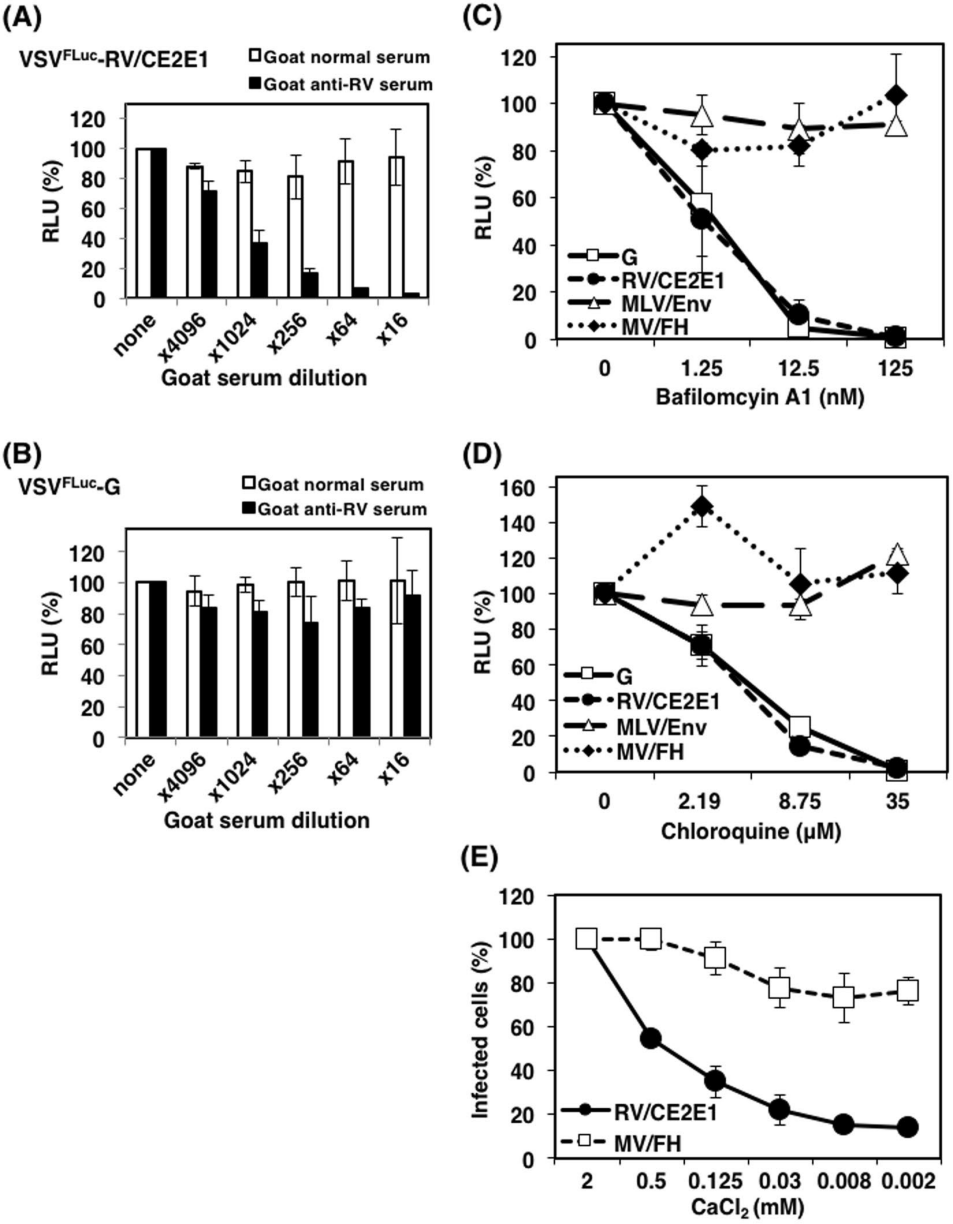
Entry mechanisms used by VSV-RV/CE2E1. Neutralization assay using goat anti-RV serum. VSV^FLuc^-RV/CE2E1 (**A**) and VSV^FLuc^-G (**B**) were incubated with the serially diluted goat antiserum against RV or with normal goat serum. Then, the Vero cells were infected with VSV^FLuc^-RV/CE2E1 or VSV^FLuc^-G pre-incubated with the serially diluted sera, and at 24 h p.i. the luciferase activities of the cells were measured. Data from VSV^FLuc^-RV/CE2E1 and VSV^FLuc^-G pre-incubated without sera were set to 100%. (**C**, **D**) Inhibition of VSV-RV/CE2E1 entry by lysomotrophic agents. Vero cells were treated with various concentrations of the following lysomotrophic agents: bafilomycin A1 (**C**), chloroquine (**D**), or were left untreated. The cells were infected with pseudotype VSVs bearing VSV, RV, MLV, or MV envelope proteins (VSV^FLuc^-G, VSV^FLuc^-RV/CE2E1, VSV^FLuc^-MLV/Env, and VSV^FLuc^-MV/FH, respectively). At 24 h p.i., the luciferase activities of the cells were measured. The luciferase activities of the cells not treated with lysomotrophic agents were set to 100%. (**E**) Ca^2+^ dependency of VSV^GFP^-RV/CE2E1 infections. Vero cells were incubated with VSV^GFP^-RV/CE2E1 or VSV^GFP^-MV/FH at 4 °C for 2 h. After washing with PBS, the cells were incubated in DMEM containing various concentrations of CaCl_2_ at 37 °C for 1 h. Next, the cells were incubated further for 24 h in standard DMEM containing 2 mM CaCl_2_, and the cell infectious units (CIUs) of the cells were determined. The CIU of the cells cultured continuously with 2 mM CaCl_2_ was set to 100%. (**A**–**G**) Data represent the mean values ± SD for triplicate samples. (**A**–**D**) RLU, relative light unit of luciferase activity.

Infection of VSV^FLuc^-RV/CE2E1, but not VSV^FLuc^-G, which has the VSV-G protein, was inhibited by anti–RV serum in a dose–dependent manner (Fig. 3A, B), confirming the functional contribution of RV proteins in VSV^FLuc^-RV/CE2E1 infections. RV enters cells through receptor–binding-mediated endocytosis and subsequent low pH–triggered viral–host membrane fusion^34–36^. To analyze the entry process of VSV^FLuc^-RV/CE2E1, the effects of endosomal acidification inhibitors on VSV^FLuc^-RV/CE2E1-infections were examined. Vero cells were pretreated with various concentrations of bafilomycin A1 and chloroquine, and infected with VSV^FLuc^-RV/CE2E1 and three control pseudotype VSVs bearing envelope proteins of VSV, murine leukemia virus (MLV), and measles virus (MV) (VSV^FLuc^-G, VSV^FLuc^-MLV/Env, and VSV^FLuc^-MV/FH, respectively). Similar to RV, entry of the authentic VSV occurs in endosomes under low pH conditions^37^, while MLV and MV enter cells in the plasma membrane under neutral pH^38–40^. As expected, the inhibitors blocked VSV^FLuc^-G infections in a dose-dependent manner, but did not block VSV^FLuc^-MLV/Env and VSV^FLuc^-MV/FH infections (Fig. 3C, D). The effect of the inhibitors on VSV^FLuc^-RV/CE2E1 was similar to that on VSV^FLuc^-G. Another feature of RV entry is its Ca^2+^ dependency^35^. Hence, the Ca^2+^ requirements of VSV^GFP^-RV/CE2E1 infection were assessed. The number of VSV^GFP^-RV/CE2E1-infected cells declined severely when the level of CaCl_2_ in the culture media was lowered (Fig. 3E). VSV^GFP^-MV/FH infectivity, which was used as a control, was reduced by only ~20% at a maximum. All these data suggest that VSV^GFP^-RV/CE2E1 undergoes a similar entry process to the authentic RV.

### Human non-immune cells are generally susceptible to VSV-RV/CE2E1, whereas immune cells are much less susceptible than non-immune cells

**Figure 4:**
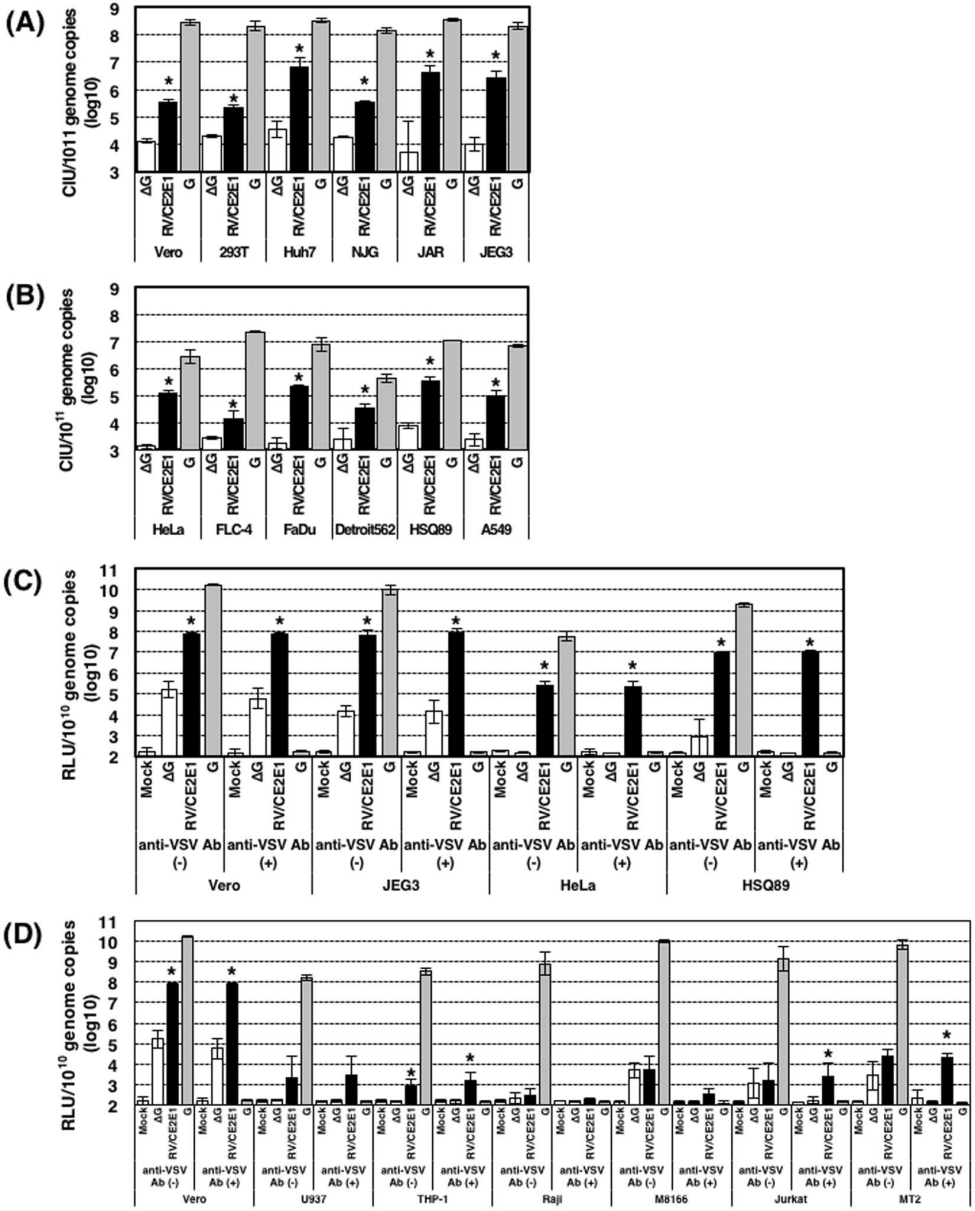
Infectivity titers of VSV-RV/CE2E1 in various cell lines. The cell infectious units (CIUs) of stock solutions of VSV^GFP^-∆G, VSV^GFP^-RV/CE2E1, and VSV^GFP^-G were determined using Vero cells and various human cell lines (**A**, **B**). The relative light unit (RLU) of stock solutions of VSV^FLuc^-∆G, VSV^FLuc^-RV/CE2E1, and VSV^FLuc^-G were determined using Vero and various human cell lines (**C**, **D**). The genome copy numbers in the stock solutions were also determined by reverse transcription-quantitative PCR, and the CIUs per 10^11^ copies and the RLU per 10^10^ copies of the viral genomes are shown. The significant differences were determined by two-tailed *t*-tests. Asterisks show significant differences with *p*-value (*P*) of < 0.05. (**A**) Data using non-immune cell lines, in which VSV^GFP^-G displayed high infectivity titers (over 10^8^ CIU/10^11^ genome copies). Vero cells, *P* < 0.0001; 293T cells, *P* < 0.0001; Huh7 cells, *P* = 0.0013; NJG cells, *P* < 0.0001; JAR cells, *P* = 0.0126; JEG3 cells, *P* = 0.002. (**B**) Data using non-immune cell lines, in which VSV^GFP^-G displayed reduced infectivity titers (under 10^8^ CIU/10^11^ genome copies). HeLa cells, *P* < 0.0001; FLC-4 cells, *P* = 0.0207; FaDu cells, *P* < 0.0001; Detroit562 cells, *P* = 0.0085; HSQ89 cells, *P* = 0.0001; A549 cells, *P* = 0.0006. (**C**) Data using non-immune cell lines with pseudotype VSVs encoding the FLuc gene treated with or without anti-VSV G antibody (anti-VSV Ab (+) and anti-VSV Ab (−), respectively). Vero cells without anti-VSV G, *P* = 0.0003; with anti-VSV G, *P* = 0.0004; JEG3 cells without anti-VSV G, *P* < 0.0001; with anti-VSV G, *P* = 0.0004; HeLa cells without anti-VSV G, *P* < 0.0001 with anti-VSV G, *P* < 0.0001; HSQ89 cells without anti-VSV G, *P* = 0.0013; with anti-VSV G, *P* < 0.0001. (**D**) Data using immune cell lines with pseudotype VSVs encoding the FLuc gene treated with or without anti-VSV G antibody (anti-VSV Ab (+) and anti-VSV Ab (−), respectively). Vero cells without anti-VSV G, *P* = 0.0003; with anti-VSV G, *P* = 0.0004; U937 cells without anti-VSV G, *P* = 0.1670; with anti-VSV G, *P* = 0.0916; THP-1 cells without anti-VSV G, *P* = 0.0149; with anti-VSV G, *P* = 0.0122; Raji cells without anti-VSV G, *P* = 0.5405 with anti-VSV G, *P* = 0.1091; M8166 cells without anti-VSV G, *P* = 0.9232; with anti-VSV G, *P* = 0.0853; Jurkat cells without anti-VSV G, *P* = 0.0843; with anti-VSV G, *P* = 0.0376; MT2 cells without anti-VSV G, *P* = 0.1080; with anti-VSV G, *P* < 0.0001. (**A**–**D**) The averages from three independent experiments are presented. Error bars indicate ± SD. ∆G, VSV-∆G; RV/CE2E1, VSV-RV/CE2E1; G, VSV-G

We analyzed VSV-RV/CE2E1, VSV-G, and VSV-∆G for their infectivity levels in various human cell lines and Vero cells. To compare the different pseudotype viruses, the infectivity titers were standardized by the genome copy numbers in the virus stocks. The genome copy numbers in VSV^GFP^-RV/CE2E1, VSV^GFP^-G, and VSV^GFP^-∆G stocks used in the experiments were 5.02 × 10^10^, 1.15 × 10^11^, and 4.29 × 10^10^, respectively, per milliliter. Also the genome copy numbers in VSV^FLuc^-RV/CE2E1, VSV^FLuc^-G, and VSV^FLuc^-∆G stocks used in the experiments were 9.35 × 10^9^, 2.81 × 10^10^, and 1.21 × 10^10^, respectively, per milliliter. VSV-G infectivity titers were generally much higher than those of VSV-RV/CE2E1 (Fig. 4). Not surprisingly, these data suggest that RV envelope proteins were less efficiently incorporated and/or less functional in VSV-based pseudotype virions than the authentic VSV G glycoprotein. Nonetheless, the VSV^GFP^-RV/CE2E1 infectivity titers were significantly higher than VSV^GFP^-∆G in many cell lines (Fig. 4). VSV^GFP^-G infectivity titers in 293T, Huh7, NJG, JAR, and JEG3 cells were similar to that in Vero cells (Fig. 4A), as were the infectivity titers of VSV^GFP^-RV/CE2E1 in 293T and NJG cells in Vero cells (Fig. 4A). In contrast, the infectivity titers of VSV^GFP^-RV/CE2E1 in Huh7, JAR, and JEG3 cells were ~10-times greater than they were in Vero cells (Fig. 4A). Vero cells are commonly used for propagating RV and it is well accepted that this cell line is susceptible to RV. These data therefore suggest that these non-immune cell lines (293T, Huh7, NJG, JAR, and JEG3) are similarly or more highly susceptible to RV than Vero cells. VSV^GFP^-G infectivity titers were reduced by 10 to 1,000-fold in HeLa, FLC-4, FaDu, Detroit562, HSQ89, and A549 cells, when compared with the titer in Vero cells (Fig. 4A, B). Therefore, these cell lines may be less susceptible or less permissible to VSV infection. Even when considering these observations, the infectivity titers of VSV^GFP^-RV/CE2E1 in these cells were significantly higher than those of VSV^GFP^-∆G (Fig. 4B), suggesting that these cell lines are also susceptible to RV. Similar experiments were performed using VSV^FLuc^-RV/CE2E1, of which the infection was highly sensitively quantified by the luciferase activity. In these experiments a VSV G protein-specific antibody, which neutralized VSV^FLuc^-G infection efficiently, was used to eliminate the possible effect by the residual VSV^FLuc^-G used for the production of VSV^FLuc^-RV/CE2E1. Data with VSV^FLuc^-RV/CE2E1 and the VSV-G neutralizing antibody confirmed the negligible or small effect by the residual VSV^FLuc^-G and the high susceptibility of non-immune cells to VSV^FLuc^-RV/CE2E1 (Fig. 4C).

In contrast, VSV^GFP^-RV/CE2E1 infectivity titers remained as low as those of VSV^GFP^-∆G in immune cell lines, and the infectivity titer, if any, of VSV^GFP^-RV/CE2E1 could not be detected in most immune cell lines (data not shown). To assess the susceptibility of the immune cell lines to VSV-RV/CE2E1 more sensitively, they were infected with VSV^FLuc^-RV/CE2E1, and any luciferase activity by the residual VSV^FLuc^-G was eliminated by the VSV G protein-specific antibody (Fig. 4D). As was expected, the infectivity titers of VSV^FLuc^-RV/CE2E1 in immune cells were undetectable in many immune cell lines (Fig. 4D). However, significant, but very low, levels of infectivity titers were detected in THP-1, Jurkat, and MT2 cells (Fig. 4D).

### Stimulation of immune cells by phorbol 12-myristate 13-acetate (PMA) increases the susceptibility of immune cells to VSV-RV/CE2E1

**Figure 5:**
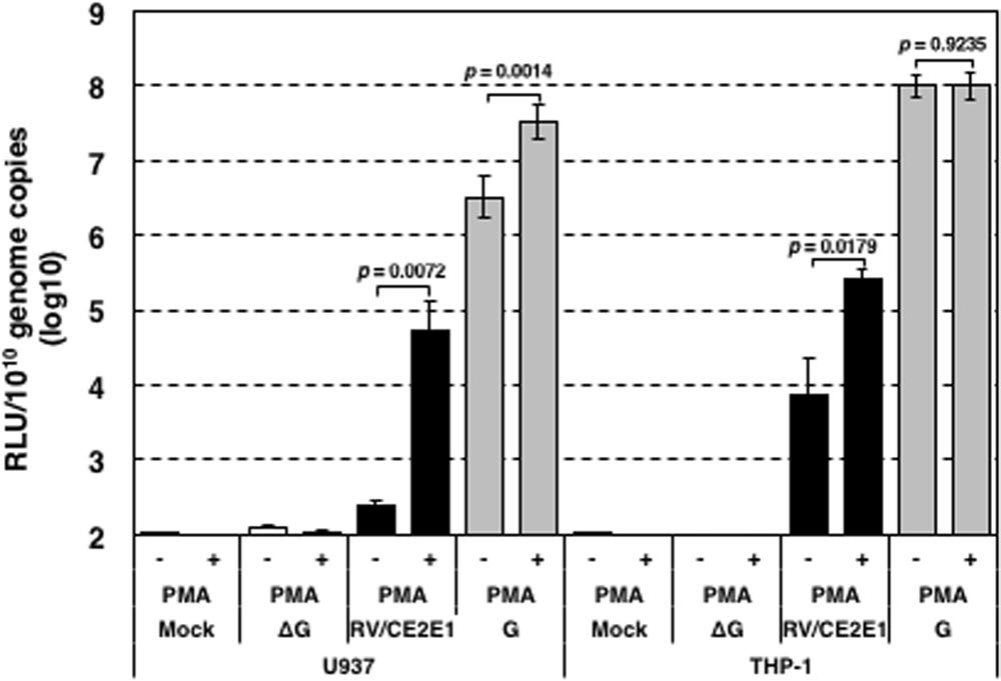
Infectivity titers of VSV-RV/CE2E1 in U937 and THP-1 cells stimulated with PMA. U937 and THP-1 were incubated with or without PMA at 37 °C for 72 h and then were infected with the pseudotype viruses. At 24 h p.i. the luciferase activities of the cells were measured. The genome copy numbers in the stock solutions were determined by reverse transcription-quantitative PCR, the RLU per 10^10^ copies of the viral genomes are shown. Data represent the mean values ± SD for triplicate samples. The significant differences were determined by two-tailed *t*-tests. RLU, relative light unit of luciferase activity; ∆G, VSV-∆G; RV/CE2E1, VSV-RV/CE2E1; G, VSV-G.

The above data demonstrated that immune cells are much less susceptible to RV than non-immune cell lines. However, previous studies demonstrated that lymphocytes become susceptible to RV after stimulation by mitogen^41–43^. U937 and THP-1 cells, derived from histiocytic lymphoma and monocytic lymphoma, respectively, were stimulated with PMA, and then infected with VSV^FLuc^-RV/CE2E1 or VSV^FLuc^-G. Unstimulated control cells were also infected with VSV^FLuc^-RV/CE2E1 or VSV^FLuc^-G. The positive effect on VSV^FLuc^-RV/CE2E1 entry by PMA stimulation was evident in THP-1 cells. The infectivity of VSV^FLuc^-G was unchanged by PMA stimulation, but the infectivity of VSV^FLuc^-RV/CE2E1 was increased by ~30-fold (Fig. 5). Similar effect was also observed in U937 cells. The infectivity of VSV^FLuc^-G and VSV^FLuc^-RV/CE2E1 were increased by ~10-fold and ~200-fold, respectively (Fig. 5). Therefore, the increase in VSV^FLuc^-RV/CE2E1 infection was possibly in part due to the positive effect by PMA for VSV replication. However, further increase in VSV^FLuc^-RV/CE2E1 suggested that the VSV^FLuc^-RV/CE2E1 entry by RV envelope proteins was promoted by PMA stimulation. These data suggested that immune cells become susceptible to RV infection after stimulation, although the levels are still much lower than those in non-immune cells.

## Discussion

The efficiency with which RV is able to infect different cell lines differs dramatically; however, it is not known which steps restrict RV infection in cells that do not support infection by this virus. Pseudotype viruses are useful tools for analyzing the susceptibility of specific viruses separately from their intracellular replication processes. A pseudotype virus with RV envelope proteins has been reported previously^31^. This is a lentiviral pseudotype virus based on the simian immunodeficiency virus (SIV). However, the SIV vector failed to incorporate the intact forms of RV envelope proteins^31^. Therefore, the SIV pseudotype virus bearing modified RV envelope proteins with the cytoplasmic tail of the VSV G protein was used in the study^31^. In the present study, a new pseudotype virus bearing the intact forms of the RV envelope proteins was generated using a VSV pseudotype system. Although the E1 protein alone causes membrane fusion and supports virus entry, the E2 and C proteins play roles in RV entry. After E1 protein binding to a receptor, RV enters cells by endocytosis^44^. Exposure to the low pH environment in early endosomes induces conformational changes in the E1 and E2 proteins and membrane fusion^34, 36^ concomitantly with a solubility change in the C protein^36^. The C protein also has a supportive role in membrane fusion^31^. The mechanism of fusion enhancement by the C protein is still unclear, but the C protein likely supports the maturation or stabilizes either E2 and E1 or their interactions during intracellular transport to the cell surface^31^. As expected, co-expression of the C protein promoted the production of infectious VSV pseudotype virus with RV envelope proteins (VSV-RV/CE2E1). Infection by the novel VSV-RV/CE2E1 pseudotype virus was indeed mediated by RV envelope proteins, and the virus underwent a similar entry process to that of the authentic RV as previously reported^35, 44^.

Using the VSV pseudotype system, we have shown that human non-immune cell lines are generally susceptible, while most immune cell lines are much less susceptible to RV than non-immune cells. Therefore, inefficient infection of immune cell lines with the authentic RV is explainable in part by the poor susceptibility of these cell lines to RV. However, previous studies have demonstrated that immune cells can become infected with RV^41–43, 45, 46^. RV replicates in peripheral blood mononuclear cells (PBMCs), of which macrophages are the main target by RV^42^. Consistent with these observations, RV is isolatable from the PBMCs of patients naturally infected with RV^46^. Unstimulated lymphocytes poorly support RV infection^42^, but are able to support RV infection upon stimulation by mitogen^41–43^. Additionally, a previous study showed that Raji and Cess human B-cell lines and the U937 monocyte line all supported RV infection^45^. Our data do not necessarily contest these previous observations, because a basal level of RV infection was observed in all the immune cell lines, although the efficiency was very low. In addition, our data further demonstrated that the infection of immune cell lines was increased to a certain level by stimulation of the cells. The key point about our data is that the entry efficiency of RV was generally high in non-immune cell lines but not in immune cell lines. These observations are important in terms of understanding more about the pathology of RV.

Even among non-immune cell lines RV infection efficiency differs dramatically, although our data using a pseudotype virus system shows that non-immune cells are generally susceptible to RV. These data suggest that the permissibility to RV infection differs considerably among non-immune cell lines. One possible factor modulating the RV infectivity is the host innate immune system. RV is highly sensitive to interferon (IFN)^3, 47, 48^, and the infectivity of individual cell lines with RV is affected by their capacity for IFN production and response to IFN. Vero cells are defective in IFN production^49, 50^, and JEG3 and JAR trophoblast cell lines, which support RV infection most efficiently, are refractory to IFN^51, 52^. Therefore, the activity of innate immunity in individual cells at least partially determines the cell or tissue tropism of RV infection. CRS is a major concern in RV infections. The high infectivity of RV in trophoblast cell lines is an interesting observation that could open a door towards understanding the establishment of RV infection in the fetus after it crosses the placenta. In the fetus, persistent infection of the endothelial cells of the fetal vessels with RV is probably a cause of CRS, because it may induce vascular abnormalities, resulting in dysfunctional or abnormal development of multiple fetal organs or tissue^53^. Perelygina *et al*. have shown that human fetal endothelial cells are persistently infected with RV^54, 55^. Further studies on the cell and tissue tropism of RV and the molecular mechanisms involved in such tropism are essential if we are to understand the pathophysiology of rubella and CRS. The new RV pseudotype system established in the present study will make a positive contribution to these additional studies.

## Materials and Methods

### Cells and viruses

Sixteen non-immune (Vero, SH–SY5Y, SK–N–MC, 293T, HUEhT–1, Caco–2, HeLa, FLC–4, Huh7, FaDu, Detorit562, A549, HSQ89, NJG, JAR and JEG3) and six immune (U937, THP-1, Raji, M8166, Jurkat, and MT2) cell lines were used in this study (Table 1). The non-immune cell lines came from various origins such as epithelial cancers, neuroblastoma, endothelial and kidney cells. The Vero, SH–SY5Y, SK–N–MC, FaDu, Detorit562, JEG3 and JAR cells were purchased from the American Type Culture Collection (Manassas, VA). NJG cells were purchased from the Health Science Research Resource Bank (Osaka, Japan). HSQ89 and HUEhT-1 cell lines were provided by the RIKEN BioResource Center (Ibaraki, Japan) and the Japanese Collection of Research Bioresources Cell Bank (Osaka, Japan), respectively. A549, FLC–4, 293T, HeLa, HSQ89, Huh7 and Vero cells were maintained in Dulbecco’s modified Eagle’s medium (DMEM) (Sigma-Aldrich, St. Louis, MO), containing 10% fetal bovine serum (FBS). Detroit562, FaDu, JEG3 and SK–N–MC cells were maintained in Eagle’s minimum essential medium (MEM) (Thermo Fisher Scientific, Waltham, MA) containing 10% FBS. Caco–2 cells were maintained in MEM containing 20% FBS. JAR and all immune cell lines were maintained in RPMI–1640 (Thermo Fisher Scientific) containing 10% FBS. SH–SY5Y cells were maintained in MEM/F–12, a 1:1 mix of MEM and Ham’s F–12 (Thermo Fisher Scientific), containing 10% FBS. HEUhT–1 cells were maintained in EGM–2 BulletKit Medium (Lonza, Basel, Switzerland). The 293CD4/DSP_1–7_ and 293FT/DSP_8–11_ cells for a DSP-based fusion assay were kindly provided by Dr. Z. Matsuda (Tokyo university, Tokyo, Japan) and maintained in DMEM containing 10% FBS and 1.5 μg/ml puromycin^56, 57^.

**Table 1:** Origins and properties of the cell lines.

Cell line	Organism	Tissue (Cell) origin	Growth property
Vero	African green monkey	Kidney	Adherent
SH-SY5Y	Human	Bone marrow: neuroblastoma	Adherent
SK-N-MC	Human	Brain: neuroepithelioma	Adherent
293T	Human	Embryonic kidney	Adherent
HUEhT-1	Human	Umbilical vein: endothelium	Adherent
Caco-2	Human	Colon: colorectal adenocarcinoma	Adherent
HeLa	Human	Cervix: adenocarcinoma	Adherent
FLC-4	Human	Liver: hepatocellular carcinoma	Adherent
Huh7	Human	Liver: hepatocellular carcinoma	Adherent
FaDu	Human	Pharynx: squamous cell carcinoma	Adherent
Detroit562	Human	Pharynx: pharyngeal carcinoma	Adherent
HSQ89	Human	Maxillary sinus; squamous carcinoma	Adherent
A549	Human	Lung; carcinoma	Adherent
NJG	Human	Uterus: gestational uterine choriocarcinoma	Adherent
JAR	Human	Placenta: choriocarcinoma	Adherent
JEG3	Human	Placenta: choriocarcinoma	Adherent
U937	Human	Pleura: histiocytic lymphoma	Suspension
THP-1	Human	Blood: acute monocytic leukemia	Suspension
Raji	Human	Lymph: B lymphoblast	Suspension
M8166	Human	Blood: T cell leukemia lymphoma	Suspension
Jurkat	Human	Blood: acute T cell leukemia	Suspension
MT2	Human	Blood: T cell leukemia	Suspension

The recombinant rubella virus, rHS, derived from RVi/Hiroshima.JPN/01.03[1J] (Hiroshima) strain, was generated as described previously^33^. The rHS strain was propagated in BHK cells twice to obtain a sufficient stock for the experiments^33^.

### Antibodies

The following mouse monoclonal antibodies were used: anti-RV capsid protein (clone Sp2/0; Abcam, Cambridge, United Kingdom), anti-RV E1 (clone 2Q2070; U.S. Biologicals, Salem, MA), anti-GAPDH (clone 3H12; MBL, Aichi, Japan), normal mouse IgG (Santa Cruz Biotechnology, Santa Cruz, CA), and anti-VSV G (clone 8G5F11; Kerafast, Boston, MA). A goat antiserum against RV was produced by immunizing a goat with purified RV particles (Baylor strain), previously inactivated by UV irradiation.

### Chemicals

Bafilomycin A1 and chloroquine were purchased from Sigma-Aldrich. The stock solution of bafilomycin A1 was prepared in dimethyl sulfoxide (Nacalai Tesque, Kyoto, Japan) at a concentration of 125 µM. A 140 mM concentration of stock solution of chloroquine was prepared in deionized distilled water.

### Plasmid constructs

The expression plasmid encoding the precursor protein for the structural proteins (C, E2 and E1) of the RV Hiroshima strain (pcDNA3.1-SP/C_1–300_) has been described previously^33^. The expression plasmid encoding only E2 and E1 proteins of the RV Hiroshima strain (pcDNA3.1-E2E1) has been described previously^33^. The expression plasmids encoding the envelope proteins of VSV (pC-VSV-G), the Edmonston vaccine strain of MV (pCA7PS-Ed-H and pCXN-Ed-F), and MLV (pFBASALF) have also been described previously^13, 58–60^. pcDNA3.1-SP/C_1–300_ is hereafter referred to as pcDNA3.1-CE2E1.

### CCID_50_ infectivity titration of RV in various cell lines

A stock solution of rHS was serially diluted 4-fold. Next, monolayers of non-immune (Vero, SH–SY5Y, SK–N–MC, 293T, HUEhT–1, Caco–2, HeLa, FLC–4, Huh7, FaDu, Detorit562, A549, HSQ89, NJG, JAR and JEG3) cells in 96–well plates were cultured with the diluted rHS samples at 35 °C. After a 4 day-incubation period, the cells were fixed with 4% paraformaldehyde and permeabilized with 0.5% Triton X–100. Then, the RV-infected cells were detected by an indirect immunofluorescent assay using the mouse monoclonal antibody specific for the RV C protein and an Alexa Fluor 594-conjugated goat anti–mouse secondary antibody (Thermo Fisher Scientific). The CCID_50_ was calculated using the Spearman–Karber formulation^61, 62^. Immune (Raji, THP–1, U937, M8166, Jurkat, MT2) cells in 96–well plates (2.0 × 10^4^ cells/well) were also cultured with the 4-fold diluted rHS samples for 4 days at 35 °C, and then fixed with 4% paraformaldehyde. The fixed cells were transferred to V-bottomed 96-well plates and centrifuged to remove the 4% paraformaldehyde fixing solution. The cells were then permeabilized with 0.5% Triton X–100. After removing the permeabilizing solution, indirect immunofluorescent staining was performed using the mouse monoclonal antibody specific for the RV C protein and an Alexa Fluor 594-conjugated goat anti–mouse secondary antibody (Thermo Fisher Scientific). The cells were transferred to flat-bottomed 96-well plates to observe the RV-infected cells using a fluorescence microscope. To discriminate the fluorescent signals from the RV-infected cells from non-specific fluorescent signals, cell nuclei were counterstained with 4′,6–diamidino–2–phenylindole (Lonza).

### Pseudotype virus generation

To generate VSV^GFP^-RV/CE2E1, 293T cells, cultured in 6-well collagen-coated plates (8.0 × 10^5^ cells/well), were transfected with the pcDNA3.1-CE2E1 expression plasmid using branched polyethylenimine (Sigma-Aldrich). At 32 h posttransfection, the cells were infected with VSV^GFP^-G at a multiplicity of infection (MOI) of 3.0^13, 16, 22, 30, 63^. The VSV^GFP^-G genome lacks the G gene, which is replaced with the GFP gene^13^. On the viral envelope the VSV^GFP^-G particles contain the G protein, which is provided in *trans* using the pC-VSV-G expression plasmid^13^). The cells were washed four times with DMEM and incubated with DMEM containing 10% FBS. After 24 h, the culture supernatants containing VSV^GFP^-RV/CE2E1 were harvested and centrifuged at 10,000 × g for 5 min at 4 °C to remove the cell debris. VSV^FLuc^-RV/CE2E1 was generated similarly to VSV^GFP^-RV/CE2E1 using VSV^FLuc^-G, which encodes the FLuc gene, instead of the GFP gene^14, 28^, at a MOI of 0.3. VSV^GFP^-RV/E2E1 and VSV^FLuc^-RV/E2E1 were also generated similarly to VSV^GFP^-RV/CE2E1 and VSV^FLuc^-RV/CE2E1, respectively, using pcDNA3.1-E2E1 instead of pcDNA3.1-CE2E1. VSV^GFP^-G, a gift from Dr. M. A. Whitt (University of Tennessee, TN), and VSV^FLuc^-G were propagated as reported previously^13, 14^. VSV^GFP^-∆G and VSV^FLuc^-∆G, which lack envelope proteins, were generated using pcDNA3.1 + (Thermo Fisher Scientific) instead of pcDNA3.1-CE2E1^13, 14^. VSV^FLuc^-MLV/Env was generated similarly to VSV^FLuc^-RV/CE2E1 using pFBASALF^58^ instead of pcDNA3.1-CE2E1. VSV^GFP^-MV/FH and VSV^FLuc^-MV/FH were also generated similarly to VSV^GFP^-RV/CE2E1 and VSV^FLuc^-RV/CE2E1, respectively, using pCXN-Ed-F and pCA7PS-Ed-H^30, 59, 60^ instead of pcDNA3.1-CE2E1. To generate VSV^GFP^-MV/FH and VSV^FLuc^-MV/FH, a fusion-blocking peptide (Z-D-Phe-Phe-Gly) (The Peptide Institute, Osaka, Japan) was used to inhibit syncytium formation during the production of these pseudotype viruses, as reported previously^30, 59^. VSVΔG/GFP-*G^63^, VSVΔG/Luc-*G^14^, VSVΔG/GFP^63^, VSVΔG/Luc^14^, MLVpv^63^ and VSVΔG*-EdHF^30^ are hereafter referred to as VSV^GFP^-G, VSV^FLuc^-G, VSV^GFP^-∆G, VSV^FLuc^-∆G, VSV^FLuc^-MLV/Env and VSV^GFP^-MV/FH, respectively, to unify the descriptions of the various pseudotype viruses.

### Electron microscopy image of viral particles

The stock solution of VSV^GFP^-RV/CE2E1 was centrifuged at 1,500 × g for 10 min at 4 °C twice to remove cell debris. The viruses were collected by centrifugation at 120,000 × g for 1 h at 4 °C onto 20% (wt/vol) sucrose cushions^9^. The pellets were resuspended in phosphate-buffered saline (PBS). The ultracentrifugation step was repeated. After fixation with 2% paraformaldehyde in PBS, the viruses were placed on a carbon-coated grid for 45 s, rinsed with distilled water, stained with 2% phosphotungstic acid solution and observed with an electron microscope (TEM-1400, JEOL, Tokyo) operating at 80 kV as previously reported^64^.

### Flow cytometry for detection of the E1 protein

293T cells were transfected with pcDNA3.1-CE2E1 or pcDNA3.1-E2E1 expression plasmids. At 36 h posttransfection, the cells were dissociated with Trypsin-EDTA (Thermo Fisher Scientific) and fixed with 4% paraformaldehyde in PBS. After washing with PBS, the cells were reacted with anti-RV E1 antibody or normal mouse IgG as a negative control. After washing with PBS containing 2% FBS three times, the cells were reacted with Alexa Flour 488-conjugated secondary anti-mouse antibody. The stained cells were detected by using a BD FACS CANTO II (Becton Dickinson, Franklin Lakes, NJ). The percentage of cells positive for the E1 protein and the mean fluorescent intensity of cells expressing the E1 protein were analyzed using a FlowJo software (Tree Star, Ashland, OR).

### Immunoblotting for detection of the E1 protein

293T cells were transfected with pcDNA3.1-CE2E1 or pcDNA3.1-E2E1 expression plasmids. At 36 h posttransfection, the cells were analyzed by immunoblotting using anti-RV E1 or anti-GAPDH antibody as previously reported^33^. The signal intensity of the E1 protein was normalized by that of GAPDH.

### A DSP-based fusion assay

A DSP-based-fusion assay was performed as described previously^56, 57^. A fusion protein of *Renilla* luciferase and GFP was split into two fragments designated dual split proteins, DSP_1–7_ and DSP_8–11_^56^. Although each fragment lacks the activities as *Renilla* luciferase and GFP, they reassemble and become functional when they are expressed within an identical cell simultaneously. 293CD4/DSP_1–7_ and 293FT/DSP_8–11_ cells constitutively expressing DSP_1–7_ and DSP_8–11_, respectively, were mixed and cultured together, and transfected with pcDNA3.1-CE2E1, pcDNA3.1-E2E1 or an empty pcDNA3.1 vector. At 32 h posttransfection, the cells were incubated with a low pH (pH5.1) culture media for 15 min to induce a cell-to-cell fusion by RV envelope proteins followed by the incubation with a standard culture media for 8 hours at 37 °C. Then the *Renilla* luciferase activity derived from the reassembled DSPs by a cell fusion were measured using the *Renilla* Luciferase Assay System (Promega, Madison, WI) and GloMax 20/20 Luminometer (Promega). The expression levels of *Renilla* luciferase were normalized by the total expression levels of E1 proteins detected by an immunoblotting as described above.

### Sucrose gradient fractionation analysis of pseudotype viruses

The pseudotype virus particles from VSV^FLuc^-RV/CE2E1 and VSV^FLuc^-G in 10 ml stock solutions were precipitated using PEG–it Virus Precipitation Solution (System Biosciences, Mountain view, CA), and the precipitated virus particles were then resuspended in 0.5 ml of DMEM. RVLP, which have been reported previously^33^ were generated and also precipitated and resuspended in 0.5 ml of DMEM, as were VSV^FLuc^-RV/CE2E1 and VSV^FLuc^-G. The resuspended VSV^FLuc^-RV/CE2E1, VSV^FLuc^-G and RVLP were layered onto 12 ml of a 20 to 40% (wt/vol) sucrose density gradient and fractionated by ultracentrifugation in an SW41 rotor (Beckman Coulter, Tokyo, Japan) at 145,000 × *g* for 2 h. Each 1 ml of the fractionated samples in the gradient was collected from the top to the bottom, and a small part of each fraction was diluted with an equal volume of DPBS. Next, the diluted samples were inoculated into the Vero cell culture media in the 96-well plates. For VSV^FLuc^-RV/CE2E1 and VSV^FLuc^-G, the RLU from the cells was detected after 24 h of incubation using the Bright–Glo Assay System (Promega) and POWER SCAN HT (BioTek, Winooski, VT), after which RLU was used as an indicator of infection with the pseudotype viruses. For RVLP, the RLU from the cells was detected after a 72 h incubation period using the *Renilla* Luciferase Assay System and GloMax 20/20 Luminometer, after which RLU was again used as an indicator of infection with RVLP.

### Neutralization assays for VSV^FLuc^-RV/CE2E1 and VSV^FLuc^-G

Goat antiserum against RV and an unimmunized normal goat serum were 4-fold serially diluted in DMEM. VSV^FLuc^-RV/CE2E1 and VSV^FLuc^-G, which express FLuc, were mixed with the diluted sera and incubated for 1 h at 4 °C. Then, the Vero cell monolayers in 96–well plates were infected with VSV^FLuc^-RV/CE2E1 and VSV^FLuc^-G pretreated with the sera. At 24 h p.i., the RLU from the cells was measured using the Bright–Glo Assay System and POWER SCAN HT.

### Analysis of the effects of endosomal acidification inhibitors on pseudotype virus infections

Vero cells were treated with various concentrations of endosomal acidification inhibitors, bafilomycin A1 or chloroquine for 30 min at 37 °C. The treated cells were infected with pseudotype viruses (VSV^FLuc^-RV/CE2E1, VSV^FLuc^-G, VSV^FLuc^-MLV/Env, and VSV^FLuc^-MV/FH), and the RLUs from the cells were detected at 24 h p.i. using the Bright–Glo Assay System and POWER SCAN HT.

### Analysis of calcium dependency in pseudotype virus infections

Calcium dependency in the pseudotype virus infections was assessed using a method similar to that reported previously^35^. Vero cells in 96-well plates were incubated with VSV^GFP^-RV/CE2E1 and VSV^GFP^-MV/FH, which express GFP, for 2 h at 4 °C, and then washed with pre–chilled DPBS, twice. The cells were then cultured in DMEM free from CaCl_2_ (Thermo Fisher Scientific) or containing various concentrations of CaCl_2_ for 1 h at 37 °C. The cells were then cultured in DMEM containing 2 mM CaCl_2_ and 10% FBS at 37 °C. At 24 h p.i. the number of GFP-expressing cells, which correspond to VSV^GFP^-RV/CE2E1- or VSV^GFP^-MV/FH-infected cells, was counted using a fluorescence microscope.

### Infectivity titration of VSV-RV/CE2E1, VSV-G, and VSV-∆G in various cell lines

Stock solutions of VSV^GFP^-RV/CE2E1, VSV^GFP^-G, and VSV^GFP^-∆G were serially diluted 10-fold. Then, monolayers of non-immune (Vero, SH–SY5Y, SK–N–MC, 293T, HUEhT–1, Caco–2, HeLa, FLC–4, Huh7, FaDu, Detorit562, A549, HSQ89, NJG, JAR and JEG3) cells in 96–well plates were infected with the VSV^GFP^-RV/CE2E1, VSV^GFP^-G, and VSV^GFP^-∆G samples at 37 °C. At 24 h p.i., the GFP-expressing cell numbers were counted. Immune (Raji, THP–1, U937, M8166, Jurkat, and MT2) cells in 96–well plates (5.0 × 10^4^ cells/well) were also infected with the 10-fold serially diluted VSV^GFP^-RV/CE2E1, VSV^GFP^-G, and VSV^GFP^-∆G samples at 37 °C. At 24 h p.i., the number of GFP-expressing cells was counted. The number was expressed in CIUs. For titration of pseudotype viruses expressing FLuc, VSV^FLuc^-RV/CE2E1, VSV^FLuc^-G, and VSV^FLuc^-∆G were incubated with anti-VSV G antibody at 4 °C for 90 min to inhibit the pseudotype virus infection by residual G proteins. Monolayer of non-immune cells (Vero, JEG3, HeLa, and HSQ89 cells) and immune cells (5.0 × 10^4^ cells/well) in 96-well plates were infected with VSV^FLuc^-RV/CE2E1, VSV^FLuc^-G, and VSV^FLuc^-∆G at 37 °C, and the RLU from the cells was detected after 20 h of incubation using the Bright–Glo Assay System and GloMax 20/20 Luminometer.

### Analysis of the pseudotype virus infectivity in stimulated U937 and THP-1 cells

U937 and THP-1 cells (3.0 × 10^4^ cells/well) in 96–well plates were incubated with or without 25 nM and 16 nM PMA (Sigma), respectively, for 72 h. VSV^FLuc^-RV/CE2E1, VSV^FLuc^-G, and VSV^FLuc^-∆G were reacted with anti-VSV G antibody at 4 °C for 90 min, and then the stimulated cells were infected with those viruses at 37 °C. The luciferase activity from the cells was detected after 20 h of incubation using the Bright–Glo Assay System and GloMax 20/20 Luminometer.

### Genome copy number measurements for VSV^GFP^-RV/CE2E1, VSV^GFP^-G, and VSV^GFP^-∆G

The genome copy number for VSV^GFP^-RV/CE2E1, VSV^GFP^-G, and VSV^GFP^-∆G was analyzed by reverse transcription-quantitative PCR (RT-qPCR) using a set of primers specific for the VSV N gene, as reported previously^19^.

### Statistical analysis

Two-tailed *t*-tests were used to determine significant differences among pseudotype virus infectivity titers in various cell lines.

### Data Availability

The datasets analyzed during the current study are available from the corresponding author on reasonable request.

## Electronic supplementary material

Supplementary Info
