# Supplementary material for: Analysis of VSV pseudotype virus infection mediated by rubella virus envelope proteins
Source: Sci Rep. 2017 Sep 14;7:11607. doi: 10.1038/s41598-017-10865-2 (PMC5599607; doi:10.1038/s41598-017-10865-2)
Supplement: Supplementary file 1 — Supplementary Info [file 41598_2017_10865_MOESM1_ESM.doc]

Title; **Analysis of VSV pseudotype virus infection mediated by rubella virus envelope proteins**

Masafumi Sakata, Hideki Tani, Masaki Anraku, Michiyo Kataoka, Noriyo Nagata, Fumio Seki, Maino Tahara, Noriyuki Otsuki, Kiyoko Okamoto, Makoto Takeda, and Yoshio Mori

**Supplementary Fig. 1.** **Infectivity titers of RV in cell lines with the NH_4_Cl.** The infectivity titer (50% cell culture infectious dose) of a stock solution of RV was determined using Vero and human cell lines (JEG3, HeLa, and HSQ89 cells). A stock solution of rHS was serially diluted 4-fold. Next, monolayers of the cells in 96–well plates were cultured with the diluted rHS samples at 35˚C. After 8 h incubation period, the samples were replaced with each fresh culture medium containing 0mM or 20mM NH_4_Cl. After 4 days incubation period at 35 ˚C, the cells were fixed with 4% paraformaldehyde and permeabilized with 0.5% Triton X–100. Then, the RV-infected cells were detected by an indirect immunofluorescent assay using the mouse monoclonal antibody specific for the RV C protein and an Alexa Fluor 594-conjugated goat anti–mouse secondary antibody. The 50% cell culture infectious dose (CCID_50_) was calculated using the Spearman–Karber formulation. Data represent the mean values ± standard deviation (SD) of three independent experiments. The significant differences were determined by two-tailed *t*-tests.

**Supplementary Fig. 2. Full-length images of immunoblotting shown in Fig. 2D.** The total amount of the E1 protein in cells co-expressed with or without the C protein. 293CD4/DSP_1–7_ and 293FT/DSP_8–11_ cells constitutively expressing DSP_1–7_ and DSP_8–11_, respectively, were mixed and cultured together. For a DSP-based fusion assay, the cells were transfected with pcDNA3.1-E2E1, pcDNA3.1-CE2E1 or the empty vector and incubated for 32 hours. The images of immunoblotting using anti-RV E1 (A) and anti-GAPDH antibodies (B), respectively. The signal intensity of the E1 protein in the cells was normalized by those of GAPDH.
